# Bias caused by sample selection for lower respiratory tract microbiome research

**DOI:** 10.1186/s13054-024-04934-y

**Published:** 2024-05-02

**Authors:** Mingqiang Wang, Xiaojie Li

**Affiliations:** 1 Zhengzhou University People’s Hospital, Henan University ‘Hospital, Zhengzhou, China; 2 Department of Critical Care Medicine, Henan Key Laboratory for Critical Care Medicine, Zhengzhou Key Laboratory for Critical Care Medicine, Henan Provincial People’s Hospital, Zhengzhou, China; 3 Department of Critical Care Medicine, The First People’s Hospital of Pinghu, Pinghu, China

Dear Editor,

We read the article by Imbert et al. [1] published on Critical Care with great interest. The authors described the characteristics of the lower respiratory tract microbiota (specifically bacteria and fungi) in patients with ARDS caused by COVID-19, influenza A, and bacterial pneumonia. Here, we offer two suggestions for the authors to consider.

Firstly, we have doubts about whether endotracheal aspirates (ETAs) can characterize the lower respiratory tract microbiota of these diseases. COVID-19 and influenza A virus primarily cause interstitial pneumonia. Over the past three years, we have encountered numerous COVID-19 patients, and a common characteristic among them is the minimal presence of sputum, particularly in cases of pure viral infection, which becomes even more apparent after negative fluid balance. Consequently, we often say that ARDS caused by COVID-19 is not typical ARDS, as these patients exhibit minimal alveolar exudation [2]. In this scenario, obtaining ETA specimens from COVID-19 patients may not represent the majority of typical COVID-19 cases. These ETA specimens could either be: 1. upper respiratory tract contaminants aspirated into the lower respiratory tract rather than originating from the lower respiratory tract; or 2. patients who not only have COVID-19 but also concurrent other infections. Here, we recommend bronchoalveolar lavage fluid as the preferred sample for studying the lower respiratory tract microbiota, as outlined in the design of an ongoing prospective, multicenter study (NCT06114784).

Secondly, there is another bias in the sample collection process of this study. The COVID-19 samples for this study were collected during the early stages of the pandemic in 2020. At that time, the sample collection and processing procedures strictly adhered to sterile protocols, including inactivation of the viruses (which seems less likely to be implemented in bacterial pneumonia). It is unclear whether the processing of ETA samples for COVID-19 in this study was the same as for other ETA samples. Different processing procedures for lower respiratory tract samples with very low microbial content can significantly affect the conclusions of the experiments.

We believe that if the author could answer or partially answer these two questions, it would enhance the credibility of this study. High quality samples are the guarantee of obtaining reliable results. Meanwhile, we suggest that researchers need to carefully consider the potential biases caused by sample types and sample processing.

## Data Availability

No datasets were generated or analysed during the current study.
